# Thoracic Osteomyelitis and Eustachian Valve Endocarditis: A Case Report and Literature Review

**DOI:** 10.7759/cureus.13810

**Published:** 2021-03-10

**Authors:** Sheila D Hernandez, Maxwell J Jabaay, Dario A Marotta, Sebastian T Tosto, Arash Velayati

**Affiliations:** 1 Internal Medicine Residency Program, Southeast Health, Dothan, USA; 2 Department of Research, Alabama College of Osteopathic Medicine, Dothan, USA; 3 Department of Neurology, Division of Neuropsychology, University of Alabama, Birmingham, USA

**Keywords:** osteomyelitis, endocarditis, eustachian valve, embryological remnant

## Abstract

Infective endocarditis and vertebral osteomyelitis are rare infections, most commonly caused by methicillin-sensitive *Staphylococcus aureus *(MSSA). The eustachian valve is an embryological remnant of the inferior vena cava that has the potential to harbor a nidus leading to infective endocarditis. Eustachian valve endocarditis has been documented in the literature on less than 50 occasions and has yet to be documented in the presence of concomitant vertebral osteomyelitis. In this case, we present a 43-year-old male presenting with vertebral osteomyelitis caused by methicillin-resistant *Staphylococcus aureus *(MRSA). Persistent bacteremia prompted the identification of vegetative growth on a eustachian valve remnant. This case helps mend the gap in the literature by documenting the treatment considerations in a patient with eustachian valve endocarditis in the presence of osteomyelitis caused by MRSA.

## Introduction

Infective endocarditis (IE) and osteomyelitis are rare and potentially life-threatening infections. *Staphylococcus aureus* (*S. aureus*) is the most common pathogen associated with both osteomyelitis and IE, with a proclivity of infections resulting from methicillin-sensitive strains [1-3]. Concomitant vertebral osteomyelitis and IE have been documented in the literature but in only about 10% of osteomyelitis cases [4]. In IE, aberrations in valvular structure often contribute to nidus formation. This fosters a bacteria-rich environment that can seed secondary infections throughout the body. Spinal dissemination of endocarditis infections most commonly arise in the lumbar spine, with a minority of cases involving cervical and thoracic vertebrae [1].

Instances of embryological heart remnants contributing to endocarditis have been infrequently documented in the literature. Of those cases, less than 50 involve an embryological remnant of the inferior vena cava (IVC), known as the eustachian valve (EV). Persistent EVs occur in approximately four percent of the general population and are generally a benign and incidental finding [5]. In the setting of prolonged bacteremia, intravenous (IV) drug use, and indwelling catheters, EV remnants have the potential to facilitate bacterial growth, most commonly with *S. aureus* [6]. To our knowledge, EV endocarditis has yet to be reported in the presence of thoracic osteomyelitis. In this case, we report a 43-year-old male presenting with encephalopathy stemming from treatment-resistant osteomyelitis of the thoracic vertebrae caused by MRSA. Further investigation revealed an elusive nidus of infection stemming from an EV remnant.

## Case presentation

A 43-year-old male presented to the emergency department via ambulance with altered mental status, visual hallucinations, and excruciating back pain as reported by an accompanying caretaker. On arrival, the patient was afebrile, mildly tachycardic (HR: 98 BPM), and borderline hypotensive (BP: 98/63 mmHg). Further examination revealed an ill-appearing, obese male in no acute distress. The patient was disoriented to place and time and was unable to follow simple commands. A detailed neurological examination could not be performed due to a lack of cooperation. The patient's vision was grossly intact and cardiovascular examination revealed a normal S1 and S2 with no murmurs, rubs or gallops. The patient had a history of poorly controlled insulin-dependent diabetes mellitus, end-stage renal disease on maintenance hemodialysis via right internal jugular vein (IJV) vascular catheter, persistent atrial fibrillation anticoagulated with apixaban, and bilateral below-the-knee amputation secondary to diabetic complications with chronic osteomyelitis from MRSA. The patient had a history of medical noncompliance and reported discontinuous medical care managed at multiple distant care facilities.

**Figure 1:**
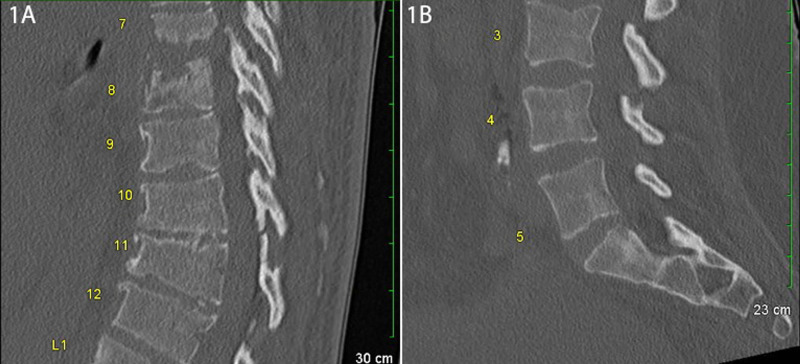
Sagittal CT of thoracic and lumbar spine. (A) Sagittal CT of the thoracic spine reveals a destructive process at T7-T8 and T10-T11 disc spaces and adjacent vertebral bodies suggestive of discitis and/or osteomyelitis. (B) Sagittal CT of the lumbar spine reveals no acute abnormalities. CT = computed tomography

A non-contrast computed tomography (CT) scan of the neuraxis revealed a destructive process in the thoracic spine at T7-T8 suspicious for discitis and osteomyelitis (Figure 1A) with unremarkable lumbar findings (Figure 1B). CT imaging of the head was negative for any acute intracranial abnormalities. Subsequent imaging studies were limited by the severity of the patient’s pain and positioning restrictions. A complete blood count was within normal limits with the exception of mild anemia (Hb: 10.1 gm/dL, Ref: 13.0-18.0 gm/dL). Complete metabolic panel revealed elevated blood urea nitrogen (41 mg/dL, Ref: 10-20 mg/dL), creatinine (3.99 mg/dL, Ref: 0.6-1.2 mg/dL), and lactic acid (3.0 mmol/L, Ref: 0.5-2.2 mmol/L). The patient did not meet sepsis criteria on arrival and was admitted for possible vertebral osteomyelitis or renal dialysis spondyloarthropathy and was started on IV vancomycin and cefepime.

**Figure 2:**
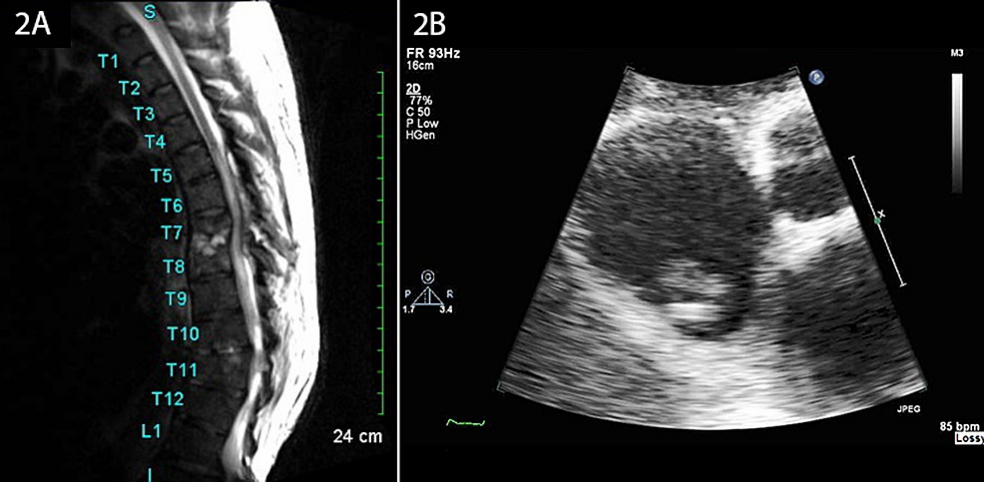
MRI of the thoracic spine and transthoracic echocardiogram. (A) Sagittal MRI of the thoracic spine confirming osteomyelitis with disc space infection at T7-T8 and similar, although much earlier, process at T10-T11. (B) An apical view of a two-dimensional transthoracic echocardiogram reveals vegetation on a eustachian valve remnant in the patient’s right atrium. MRI = magnetic resonance imaging

Magnetic resonance imaging (MRI) of the thoracic spine confirmed osteomyelitis at T7-T8 and a similar yet earlier infectious process occurring at T10-T11 (Figure 2A). Blood culture results at day 3 revealed methicillin-resistant *S. aureus* (MRSA). A CT-guided biopsy of the spinal abscess was scheduled but was later cancelled per the patient's request. The patient was managed on an in-patient basis for a total of 16 days with serial blood cultures. Repeat blood cultures at the end of week 1 remained positive for MRSA. The patient’s right IJV vascular catheter was removed and tested positive for MRSA. The catheter was replaced on two separate occasions and tested positive for MRSA both times. Vancomycin therapy was superseded by IV daptomycin (800 mg post-dialysis tri-weekly) and rifampin (300 mg twice daily) due to persistent bacteremia.

Blood cultures remained positive for MRSA at the beginning of week 2. Concerns surrounding the persistent bacteremia prompted a transthoracic echocardiogram (TTE), which revealed the presence of a mobile vegetation on an embryological remnant of the IVC, an EV remnant, within the inferior aspect of the right atrium (Figure 2B). In-patient IV daptomycin was continued through post-admission day 13 when the patient’s cognitive symptoms resolved. The patient was discharged on post-admission day 16 with arrangements to return for scheduled IV daptomycin infusions for an additional six weeks. Following antibiotic therapy, the patient has not returned for treatment related to this condition.

## Discussion

IE is a focal infection of the endocardium that has a high potential for systemic dissemination. In the United States, IE is rare and occurs at a rate of 11.4 per 100,000 adults [7,8]. Common signs of endocarditis include fever, new or increasing cardiac murmur, and embolic phenomenon, such as Olser's nodes or Janeway lesions [9]. Fever, the most commonly associated sign of endocarditis, was notably absent in this patient. Nevertheless, even if a fever was present in this patient it could have been attributed to the more evident diagnosis of osteomyelitis. This underscores the importance of a thorough evaluation, especially in patients with accompanying risk factors. IE risk factors include a past history of IV drug abuse, embedded medical hardware, an aberration in heart structure, and immunosuppression [6,10,11]. While this patient denied IV drug use, the use of a dialysis catheter in combination with immunosuppression by virtue of uncontrolled diabetes increased the overall risk of bacterial growth and subsequent spread [12].

The presence of structural heart defects and bacteremia should raise the index of suspicion for IE, but not all defects are outwardly evident. In this case, the patient did not display overt signs of structural heart defects, such as an audible murmur or patient-reported diagnosis. A broad screening effort included the use of a TTE to rule out the heart as a source of persistent infection. While TTE is less specific for right atrial pathology than transesophageal echocardiography (TEE) due to limitations of probe positioning, TTE remains the preferred initial screening modality [11,13,14]. Fortunately, the patient’s embryological EV remnant was detected on TTE as a potential source of seeding infection, thus there was no need to pursue a TEE. This remnant is present in 2%-4% of the adult population [15]. The EV functions in-utero to direct vital oxygenated blood from the IVC to the foramen ovale and then to the left atrium. After birth and closure of the foramen ovale, the EV regresses, but this regression is variable and can lead to a remnant of the valve located on the superior aspect of the IVC [15,16]. The remnant is normally of little clinical significance [16]. However, in the presence of bacteremia, the abnormality can contribute to progressive nidus formation and persistent systemic spread.

In the setting of IE, bacterial dissemination to the spine most commonly occurs in the lumbar vertebrae [1]. Vertebral osteomyelitis occurs at a rate of 22.4 per 100,000 adults and should be considered in the presence of severe back pain, cognitive disturbances, and bacteremia [17]. The link between IE and concomitant vertebral osteomyelitis can partially be explained by vascular anatomy. The posterior intercostal artery branches from the descending aorta and supplies blood to the spinal arteries. Then, the spinal arteries supply blood to the vertebrae. This pathway serves as a migratory route for the hematogenous spread of bacteria following cardiac colonization in the setting of IE. Contiguous spread to nearby organs and extension of the infection throughout the axial spine can occur. As the disease progresses, worsening bacteremia can result in a pronounced inflammatory state and attenuation of existing renal failure. Increases in interleukin-6 (IL-6) and interleukin-1β (IL-1β) can trigger iron sequestration and shorten the lifespan of erythrocytes leading to anemia as seen in this patient [18]. Further, exacerbation of renal failure can suppress excretion of neurotoxins, such as ammonia, causing cognitive deficits and encephalopathy.

Clinical treatment of coexisting IE and vertebral osteomyelitis relies on pathogen-directed therapy. In this case, the presence of MRSA in serial blood cultures limited effective therapy to narrow-spectrum IV antibiotics, such as vancomycin and daptomycin. A minimum of six weeks of therapy is recommended, with eight to 12 weeks warranted in treatment-resistant strains [19]. Nevertheless, dose adjustment in the setting of renal compromise is necessary with regular monitoring to detect complications. In this case, after careful dialogue between the patient and care team, the patient was discharged from the care facility in a stable condition with arrangements to return for scheduled IV antibiotic infusions. While this management decision may not be warranted in every clinical situation, it was made to balance the patient’s cooperation, autonomy, and perceived quality of life.

A version of this article was posted on a preprint server (Authorea) on May 6, 2020 [20]. The preprint version is not pending full publication elsewhere.

## Conclusions

Vertebral osteomyelitis in the presence of persistent or recurrent bacteremia warrants screening for active IE. This case report documents an embryological remnant of the IVC, known as the EV, as a source of IE in the setting of vertebral osteomyelitis. While this constellation of comorbidities is rare, this case underlines the importance of a thorough evaluation and persistent monitoring in the treatment of such persistent infectious conditions.
